# Origin of Diphtheria

**Published:** 1881-07

**Authors:** 


### ORIGIN OF DIPHTHERIA.

The observations of Mr. G. H. Fosbrooke, medical health officer of Birmingham, England, have led him to form conclusions respecting the origin of diphtheria which differ in some points from those which have been urged by other authorities. He regards it as a well-established fact, confirmed by his experience, that the disease is more common in rural than in urban districts, and has observed that even when it has prevailed extensively in a rural district, and has thence been conveyed into a neighboring town, it has not spread in the town. In one town of 5,000 inhabitants, diphtheria, when it occurred, prevailed concurrently with typhoid fever or scarlatina, giving rise to the suggestion that all those diseases might originate in a common poison. Mr. Fosbrooke does not agree with other authorities as to the conditions of soil most favorable to the propagation of diphtheria. Generally the disease has been thought to flourish most in damp situations and in connection with damp subsoils. All of his attempts to associate its origin and distribution with any peculiar soil or situation have failed, for he has met it both in villages occupying elevated airy situations and in low places. The most serious epidemics, and the larger number of cases of which he has had personal knowledge, have appeared on soils that were "rather gravelly and well drained." With one exception, his experience opposes the idea that houses shut in by trees are more liable to harbor the disease than those which are not surrounded by an abundant vegetation. The fluctuations of diphtheria, when it prevails for any considerable length of time, do not appear to be influenced by changes of season or by variations of weather. Meteorological observations, made with reference to this point, differ widely, and furnish no guide to an opinion. The disease is generally found first to break out in October, and to prevail as an epidemic, when it does so prevail, in the winter months, increasing, as is natural with epidemics, during the earlier months of its course, but without regard to the regularity or irregularity of the season.—*Popular Science Monthly.*

A LITTLE girl once took a letter from her mother to an old lady friend.

"Many thanks, my child," she said; "you may tell your mother that you are a good child and are a faithful little messenger."

"Thank you, ma'am; and I shall tell her, too, that I didn't ask you for ten cents, because mamma told me not to."—*French paper.*

